# Systematic literature review and narrative synthesis of the use of natural language processing to triage outpatient referrals

**DOI:** 10.3389/frhs.2026.1797583

**Published:** 2026-04-15

**Authors:** Angela Lim Fung, Mohamed Abdalla, Yu Blanche Cheng, Manar Elsayed, Dagmara Chojecki, Joseph Ross Mitchell, Carrie Ye

**Affiliations:** 1 Institute of Medical Science, University of Toronto, Toronto, ON, Canada; 2 Division of General Internal Medicine, Department of Medicine, University of Alberta, Edmonton, AB, Canada; 3 The Alberta Machine Intelligence Institute, Edmonton, AB, Canada; 4 Department of Laboratory Medicine and Pathobiology, Temerty Faculty of Medicine, University of Toronto, Toronto, ON, Canada; 5 Division of Rheumatology, Department of Medicine, University of Alberta, Edmonton, AB, Canada; 6 Institute of Health Economics, Edmonton, AB, Canada; 7 Department of Computing Sciences, University of Alberta, Edmonton, AB, Canada; 8 Arthritis Research Canada, Vancouver, BC, Canada

**Keywords:** artificial intelligence, clinical triage, machine learning, natural language processing, outpatient care, referral prioritization

## Abstract

**Background:**

Natural Language Processing (NLP) models show promise in enhancing interpretation and triage of outpatient referrals across diverse specialties.

**Objective:**

To conduct a systematic literature review and narrative synthesis of recent studies that utilized NLP-based models for triage-related tasks such as urgency prioritization, referral classification, and justification review.

**Methods:**

Medline, Embase, Web of Science, and CINAHL databases were searched for articles published up to February 17 2024, limiting searches to the last 5 years prior to the search. All citations were imported into Covidence for duplicate removal and screening. We included studies that utilized NLP techniques to triage outpatient referrals to a specialist (medical or surgical), and included comparison to human triage. Abstracts and full texts were each screened independently by two reviewers. Data from each study were extracted independently by two reviewers using a standardized extraction form, including fields such as study design, dataset size, specialty, models tested, and outcomes reported. Results were synthesized narratively, organized by key themes focused on data, model and clinical applicability. Quality and risk of bias assessment was performed using the PROBAST-AI and Technology Readiness scales.

**Results:**

A total of 4,225 titles and abstracts were reviewed resulting in 26 full-text reviews. A total of 10 studies were used for data extraction and synthesis. These studies spanned a wide range of medical specialties including surgery, medical specialties, and radiology. Tasks included predicting condition and priority level. Most domains were assessed as low or uncertain risk of bias. Outcome measures varied across studies, but overall, 7 studies reported high levels of accuracy compared to manual workflows. We summarized key differences in dataset preprocessing and augmentation, triage model, and feasibility and clinical applicability.

**Conclusion:**

NLP shows promise in augmenting human triage of outpatient referrals to specialty care. To realize the full potential of NLP for triage, future work should prioritize standardized reporting and prospective validation to support safe and effective integration into healthcare systems.

## Introduction

Effective triage systems are essential for timely processing and prioritization of outpatient referrals. In most healthcare systems, outpatient specialists require referrals from primary care providers or other specialists to initiate further evaluation, diagnosis, or treatment. Subsequent triage by the receiving specialists’ team is essential to ensure that patients are seen in a timely manner, reduce unnecessary healthcare utilization and improve patient outcomes (1, 2). However, triaging these referrals is inherently complex, as it involves prioritizing patients based on the urgency of their condition alongside constraints in specialist availability and clinical resources. Given the increasing backlog in outpatient care worldwide, there is an urgent need for novel systems to prioritize urgent referrals, reduce wait times and ultimately, improve patient outcomes (3–5).

Traditional triage processes rely on manual review of referral letters, which can be labor-intensive, time-consuming, and susceptible to variability in clinical judgment (6, 7). There is therefore a growing interest in leveraging computational methods to support, optimize, and/or automate referral triage. Referral letters are often composed of free text data and thus natural language processing (NLP) techniques, in particular, may be useful to interpret these data. NLP is a subfield of artificial intelligence (AI) focused on enabling machines to understand and interpret human language (8). Specific to outpatient triage workflows, NLP techniques can be applied to unstructured clinical text and perform functions such as classification and prioritization (9, 10). Recent advances in deep learning, particularly in transformer-based models such as BERT (Bidirectional Encoder Representations from Transformers), and large language models (LLMs) such as GPT, Claude, Gemini, and Llama, have enabled more sophisticated understanding of clinical narratives, allowing for accurate categorization of referrals (11, 12).

This systematic literature review aims to evaluate and describe recent studies utilizing NLP in AI-driven models for triage of referrals to secondary or tertiary care settings, with an emphasis on performance metrics and clinical feasibility. Specifically, the review aims to identify the types of models used (e.g., deep learning, traditional machine learning, rule-based systems), the clinical contexts in which they have been applied, and the effectiveness of these approaches compared to standard triage practices. Through this review, we seek to narratively discuss existing evidence on NLP-assisted triage, assess clinical utility of various models, and identify outpatient contexts where implementation provided maximal benefits. A glossary of terms used in this paper can be found in Box 1.

Box 1 **Glossary.**

**Table:** 

**Term**	**Explanation**
Area under the Curve (AUC)	Performance metric that assesses ability to distinguish between classes. A score of 1.0 indicates a perfect distinction.
Bag of Words (BoW)	Simplifying representation where a text is represented as a multiset of its words; grammar and word order are disregarded, but the frequency or presence of specific terms is maintained.
Bidirectional Encoder Representations from Transformers (BERT)	Deep learning model that processes words in relation to all other words in a sentence (bidirectional).
Bidirectional Long Short-Term Memory (Bi-LSTM)	Variant of a Recurrent Neural Network that processes data in both forward and backward directions.
Convolutional Neural Network (CNN)	Deep learning model used to identify local patterns within text.
Decision Tree	Supervised learning algorithm that uses a flowchart-like tree structure to reach a conclusion.
F1	Performance metric that is the harmonic mean of precision and recall.
k-Nearest Neighbors (kNN)	Algorithm that classifies a new data point based on its proximity to others in a multi-dimensional space.
Latent Dirichlet Allocation (LDA)	Method used in Topic Modelling that can detect topics within large collections of unstructured text without needing prior labels.
Logistic Regression (LR)	Statistical and machine learning model used for binary classification, that calculates the probability that a given input belongs to a specific category.
Multilayer Perceptron (MLP)	Neural network where data is processed though an input layer, hidden layers, then an output layer to reach a final classification.
Naive Bayes	Probability classifier based on Bayes’ Theorem; assumes all features are independent of each other.
Precision	Performance metric that measures the accuracy of positive predictions. Higher precision indicates fewer “false positives”. Also known as positive predictive value.
Recurrent Neural Network (RNN)	Deep learning model that processes sequential data, maintaining memory of earlier words.
Stop Words	Commonly used words (e.g."the”,"is”) that are filtered out prior to model training or prediction.
Support Vector Machine (SVM)	Supervised learning model that identifies an optimal boundary to separate different classes in a high-dimensional space.
Term Frequency-Inverse Document Frequency (TD-IDF)	An advanced variant of Bag of Words where text is represented as a multiset of its words but the representation incorporates the frequency of words in and across texts.
Tokenization	Data processing strategy where text is broken down into smaller units called tokens (usually individual words or sub-words).
Topic Modelling	Unsupervised learning task where models are used to identify hidden thematic patterns within a large collection of unstructured text.
Random Forest	Supervised learning method that builds an ensemble of multiple individual decision trees.
Recall	Performance metric that measures the ability of a model to identify all postive cases. Also known as senstivity.
Word Embeddings	Data processing strategy that converts words into numerical vectors, with words with similar meanings placed close together in a high dimensional space to represent semantic relationships.

## Methods

This study was conducted and written following the Preferred Reporting Items for Systematic Reviews and Meta-Analyses (PRISMA) guidelines (13). A protocol was developed in advance and screening and data extraction were conducted in Covidence (Veritas Health Innovation Ltd), an online platform designed to support systematic reviews. The study protocol was registered in the “International Prospective Register of Systematic Reviews” (PROSPERO) in 2024 (CRD42024586281). A refined PICOS question was constructed inquiring about performance of NLP techniques for triage of referrals to secondary to tertiary clinical settings, as compared to manual triage methods. A comprehensive search strategy was developed in collaboration with an experienced librarian (DC, Supplementary Table 1).

### Study search and screening

Medline, Embase, Web of Science, and CINAHL databases were searched for articles published up to February 17 2024, limiting searches to the last 5 years prior to the search. We chose to limit searches to the most recent 5 years as we wanted our results to reflect the rapid recent advancements in NLP. Articles were identified using a combination of controlled vocabulary (e.g., MeSH headings) and relevant keywords relating to triage and referral, artificial intelligence, natural language processing, machine learning, and large language models. Searches were limited to the English language, but not to geographic regions. All citations were imported into Covidence for duplicate removal and screening. Abstracts and full texts were each screened independently by two reviewers (AF, YC, ME). Full texts were retrieved for abstracts deemed potentially relevant and assessed against predetermined inclusion and exclusion criteria. Disagreements were resolved through a third reviewer (CY).

We included studies that utilized NLP techniques to specifically triage (label) outpatient referrals. We included studies that involved patients being referred to a specialist (medical or surgical), evaluated NLP model(s) (index model), and included comparison to conventional methods of triaging (reference standard). We excluded studies that did not pertain to referrals to specialty care (e.g., emergency department triage), did not use any NLP techniques, did not involve comparison to a conventional triaging system, and/or did not report specific outcomes. Peer reviewed journal publications, dissertations, as well as gray literature (i.e., articles not published through traditional academic channels and/or peer-reviewed) were included. Systematic reviews, meta-analyses, editorials, comments, abstracts, commentaries, correspondences, erratum, guidelines, protocols, and video articles were excluded.

### Data extraction and analysis

Data from each study were extracted independently by two reviewers (AF, YC, ME) and disagreements were resolved by consensus between two additional reviewers with expertise in clinical triage (CY), and NLP (MA). A standardized extraction form was used, including fields such as study design, dataset size, specialty, models tested, and outcomes reported. Exact values (i.e., for dataset size and allocation) were either retrieved or calculated by reviewers where possible. Performance metrics for the best performing model and results from any prospective and/or feasibility assessments were extracted. Due to heterogeneity in study designs, clinical contexts, NLP approaches, and reported outcomes, a meta-analysis was not performed. Results are presented in a qualitative format and synthesized narratively, organized by key themes focused on data, model and clinical applicability.

### Quality assessment

Two reviewers (AF, BC) independently assessed each study using the Prediction Model Risk of Bias Assessment Tool for Artificial Intelligence (PROBAST-AI) (14) and the Technology Readiness Level scale adapted for the medical domain (15), and discrepancies were resolved by a third reviewer (CY or MA).

#### Risk of bias and applicability

The quality of the included studies was evaluated using the PROBAST-AI (14). This tool rates both risk of bias and applicability of AI-based prediction models. The included studies received scores for four domains for Risk of Bias (Participants & Data Sources, Predictors, Outcome, and Analysis) and three domains for Applicability (Participants & Data Sources, Predictors, and Outcome). A rating of “Low,” “High,” or “Unclear” was assigned for each domain, and an overall rating for risk of bias and applicability was also assigned. As per PROBAST-AI guidelines, if any single domain was rated as High Concern, the overall rating was High. Similarly, if any single domain was rated as Unclear, and none of the remaining domains were rated as High Concern, the overall rating was Unclear. Model development and model evaluation were assessed separately.

#### Technology readiness

To determine the clinical readiness of the identified models, each study was assigned a Technology Readiness Level (TRL) (15). This is a 9-level scale indicating increasing clinical maturity, ranging from 1: Clinical problem identification, to 9: Model integration.

## Results

### Study selection and characteristics

**Figure 1:**
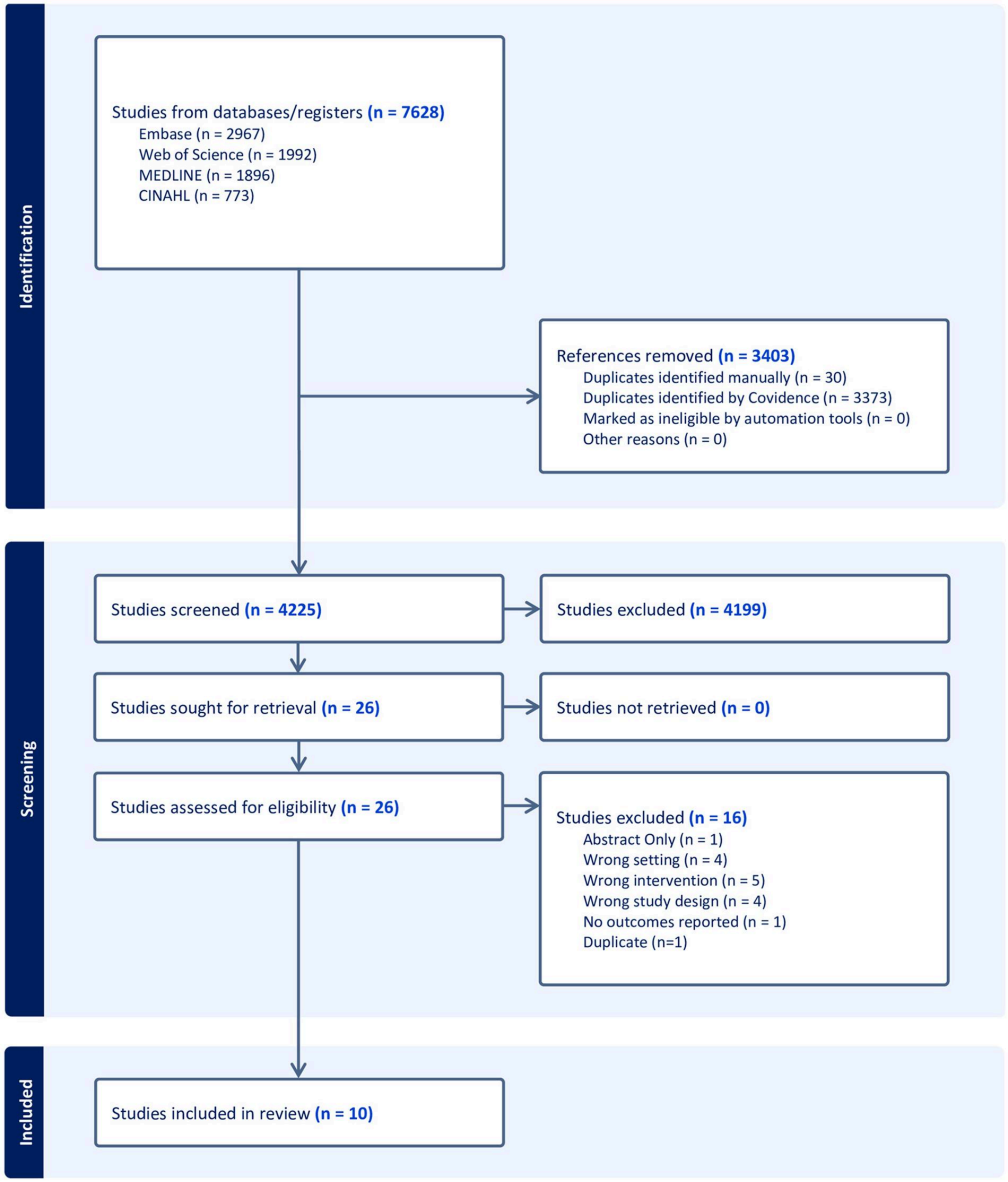
PRISMA flow diagram.

The initial database searches yielded 7,628 records and after 3,403 duplicates were removed, 4,225 titles and abstracts were reviewed. 26 full text studies were retrieved and assessed for eligibility (Figure 1). After full text review, 11 studies met criteria for data extraction, but two nearly identical studies using the same dataset were published by the same authors on the same date (16, 17), and thus only one (16) of these was included, leaving a total of 10 studies for data extraction and synthesis (Table 1) (16, 18–26). Clinical settings included ophthalmology, rheumatology, otorhinolaryngology, internal medicine, surgery, anesthesiology, radiation oncology, radiology, and rehabilitation. The included studies included data from the Netherlands, Chile, Canada, the United Kingdom, Ireland, the United States, and Australia. All studies included retrospective cohorts and two studies also included prospective validation cohorts (18, 26). The aim of these studies focused on prediction of the condition, intervention, eligibility and urgency.

**Table 1:** Summary of study characteristics.

STUDY	COUNTRY	STUDY TYPE	REFERRAL	AIM
Fudickar 2024 (20)	Netherlands	Retrospective Cohort	Back pain (Neurosurgery, Anesthesiology, Rehabilitation)	Predict intervention based on referral letters, self-report questionnaires, and electronic health records
Abdel-Hafez 2023 (21)	Australia	Retrospective Cohort	Otorhinolaryngology	Predict level of priority, condition, and associated clinical prioritization criteria based on referral letters
Wang 2023 (18)	UK	Retrospective/ Prospective Cohort	Rheumatology	Predict type of condition and associated probability based on referral letters
Aakre 2022 (22)	USA	Retrospective Cohort	Internal Medicine	Predict specialty selection based on appointment request forms
Potocnik 2022 (24)	Ireland	Retrospective Cohort	Radiology	Predict level of justification based on referral letters
Alanazi 2022 (16, 17)	Ireland	Retrospective Cohort	Radiology	Predict indication for scanning based on referral letters
Guzman 2021 (23)	UK	Retrospective Cohort	Spine Surgery	Predict level of urgency based on referral letters
Tan 2021 (25)	Australia	Retrospective Cohort	Ophthalmology	Predict level of urgency based on referral letters
Villena 2021 (26)	Chile	Retrospective/ Prospective Cohort	Medical surgical specialties	Predict coverage under Explicit Health Guarantees based on referral letters
Spasic 2020 (19)	UK	Retrospective Cohort	Musculoskeletal conditions (various services)	Assign topics relevant to prescribing treatment based on referral letters

### Quality assessment

#### Risk of bias and applicability

Of the included studies, two (18, 26) were assessed on both model development and model evaluation, as they included external validation on prospective data. The remaining studies were assessed on model development only (Table 2). For model development, 4/10 studies (16, 20, 23, 25) were rated as having an overall High Concern for risk of bias, while 4/10 (18, 19, 21, 24) were rated as unclear. Two studies (22, 26) were rated as Low Concern for risk of bias across all domains. For model evaluation, Villena et al. (26) was rated as Low Concern across all domains, while Wang et al. (18) was rated as Unclear for Risk of Bias. The latter rating was due to the prospective pilot being conducted at the same location as the source of the model development data, which does not necessarily meet criteria for external validation. In contrast to the quality concerns, all included studies demonstrated Low Concern for Applicability, as they utilized raw data that were relevant to the clinical workflows (e.g., real-world referral texts) and that directly matched targeted outcomes.

**Table 2:** PROBAST-AI.

	1. Participants & DATA SOURCES		2. PREDICTORS		3. OUTCOME		4. ANALYSIS	OVERALL	
Study	ROB	Applicability	ROB	Applicability	ROB	Applicability	ROB	ROB	Applicability
MODEL DEVELOPMENT									
Fudickar 2024 (20)	High concern	Low concern	Low concern	Low concern	Low concern	Low concern	High concern	High concern	Low concern
Abdel-Hafez 2023 (21)	Low concern	Low concern	Low concern	Low concern	Unclear	Low concern	Low concern	Unclear	Low concern
Wang 2023 (18)	Unclear	Low concern	Low concern	Low concern	Low concern	Low concern	Unclear	Unclear	Low concern
Aakre 2022 (22)	Low concern	Low concern	Low concern	Low concern	Low concern	Low concern	Low concern	Low concern	Low concern
Potocnik 2022 (24)	Unclear	Low concern	Low concern	Low concern	Low concern	Low concern	Unclear	Unclear	Low concern
Alanazi 2022 (16, 17)	Unclear	Low concern	Low concern	Low concern	Low concern	Low concern	High concern	High concern	Low concern
Guzman 2021 (23)	Low concern	Low concern	Low concern	Low concern	Low concern	Low concern	High concern	High concern	Low concern
Tan 2021 (25)	Low concern	Low concern	Low concern	Low concern	Low concern	Low concern	High concern	High concern	Low concern
Villena 2021 (26)	Low concern	Low concern	Low concern	Low concern	Low concern	Low concern	Low concern	Low concern	Low concern
Spasic 2020 (19)	Low concern	Low concern	Low concern	Low concern	Low concern	Low concern	Unclear	Unclear	Low concern
MODEL EVALUATION									
Wang 2023 (18)	Low concern	Low concern	Low concern	Low concern	Low concern	Low concern	Unclear	Unclear	Low concern
Villena 2021 (26)	Low concern	Low concern	Low concern	Low concern	Low concern	Low concern	Low concern	Low concern	Low concern

ROB, risk of bias.

#### Technology readiness

8/10 studies (16, 18, 20–25) were classified as level 4 for clinical readiness, corresponding to a model prototyping and development stage (Table 3). Most included studies developed and tested models with retrospective data. The study by Spasic and Button was rated as level 3, as it focused mainly on the feasibility of unsupervised topic modeling (19). The study by Villena et al. was classified as level 7, corresponding to the workflow implementation stage. This study's model was more advanced as it was deployed in a live hospital setting where it was used voluntarily by staff to amend human misclassifications (26).

**Table 3:** Clinical machine learning readiness.

STUDY	LEVEL
Fudickar 2024 (20)	4
Abdel-Hafez 2023 (21)	4
Wang 2023 (18)	4
Aakre 2022 (22)	4
Potocnik 2022 (24)	4
Alanazi 2022 (16, 17)	4
Guzman 2021 (23)	4
Tan 2021 (25)	4
Villena 2021 (26)	7
Spasic 2020 (19)	3

### Narrative synthesis

The included studies all applied NLP techniques to perform a range of triage tasks relevant to specialty care, aiming to develop automated tools that support triage decision-making. Table 4 summarizes the outcome measures for the best performing NLP model in each study and the datasets used for training and evaluation. We narratively synthesized three main aspects of the included studies: (1) Dataset preprocessing and augmentation; (2) NLP triage model performance; and (3) Feasibility and clinical applicability.

**Table 4:** Summary of training and test datasets and outcome measures.

STUDY	DATASET	TRAIN	TEST	TASK	MODEL NAME (BEST PERFORMING)	METRICS (BEST PERFORMING)
Fudickar 2024 (20)	Referral letters + self-report questionnaires *n* = 1,009	4-fold cross validation *n* = 1,009		Multiclass classification of intervention	SVM	F1: 0.535
Abdel- Hafez 2023 (21)	Referral letters and clinical prioritization criteria text *n* = 17,378, 9.6% labelled	5-fold cross-validation *n* = 1,689		Multiclass classification of disease and priority levels	Text similarity (Levenshtein distance)	Level of agreement: 0.538 Precision: 51.5% Accuracy: 53.8% Sensitivity: 50.9%
Wang 2023 (18)	Referral letters *n* = 1,264 Blood test results *n* = 1,181 Ensemble (both) *n* = 119	5-fold cross validation *n* = 1,264 (referral letters) *n* = 1,181 (blood test results)	Test set of ensemble *n* = 79	Binary classification of disease	MBERT and LightGBM (Weighted AUC fusion)	Precision: 0.83 Accuracy: 0.82 Recall: 0.82 F1: 0.83 G-Mean: 0.83 AUC: 0.90
Aakre 2022 (22)	Appointment request forms *n* = 8,169 (labelled) + 227,672 (pseudo-labelled)	Hyperparameter tuning *n* = 817 Final training *n* = 6,534 (labelled) + 16,384 (pseudo-labelled)	Hold-out set: *n* = 817	Multiclass classification of specialty	CNN	F1: 0.775 AUC: 0.949 MCC: 0.734 Hamming loss: 0.048
Potocnik 2022 (24)	Referral letters *n* = 375	Training set *n* = 300	Hold-out set *n* = 75	Binary classification of justification	BoW + CSW + SC + SVM	Weighted accuracy: 92.2% Recall: 91.1% AUC: 0.948 Specificity: 93.3%
Alanazi 2022 (16, 17)	Referral letters *n* = 1,020	Training set *n* = 920	Hold-out set *n* = 100	Binary classification of indication	Bi-directional neural network	Precision: 0.92 Accuracy: 0.86 Recall: 0.90 F1: 0.91 Kappa: 0.56
Guzman 2021 (23)	Referral letters *n* = 413	10-fold stratified and nested cross-validation *n* = 413		Binary classification of urgency	AdaBoost	Precision: 58.60% Accuracy: 63.90% Recall: 48.6% F1: 53.10% AUC: 61.80% Specificity: 75.0%
Tan 2021 (25)	Referral letters *n* = 208	5-fold cross-validation *n* = 156	Hold-out set *n* = 52	Binary classification of urgency; Multiclass classification of priority	CNN	Binary classification Precision: 0.9695 NPV: 0.71 Accuracy: 0.81 Recall: 0.69 Specificity: 0.96 F1: 0.80 AUC: 0.83 Multiclass classification Accuracy: 0.65
Villena 2021 (26)	Referral letters *n* = 2,105,129 (labelled) + ∼11,000,000 unlabelled + 942 expert labelled +	Training set (word embeddings) *n*=∼1,000,000 unlabelled Training set (classification) *n* = 60% of 2,105,129 labelled	Hold-out set *n* = 40% of 2,105,129 labelled Expert labelled *n* = 942	Binary classification of coverage	Random forest	Hold-out set Precision: 0.92 Recall: 0.91 F1: 0.91 AUC: 0.96 Expert labelled: Precision: 0.87 Recall: 0.86 F1: 0.85 AUC: 0.94
Spasic 2020 (19)	Referral letters *n* = 576	10-fold cross validation *n* = 576		Binary classification of multiple treatment options	LDA	Accuracy Orthopedic: 90.11% Discharge: 62.81% Injection: 80.54% Nutritionist: 97.4% Physiotherapy: 71.52% Imaging: 77.6% Surgery: 81.95% Review: 56.57% Other: 97.23% Kappa = 0.73

AUC, area under the curve; BoW, Bag of Words; CNN, convolutional neural network; CSW, custom stop words; LightGBM, light gradient boosting machine; LDA, Latent Dirichlet Allocation; LR, logistic regression; MBERT, multilingual bidirectional encoder representations from transformers; SC, spell correction; SVM, support vector machine; UMLS, Unified Medical Language System.

#### Dataset preprocessing and augmentation

Dataset preprocessing and augmentation varied significantly across studies, depending on the nature and source of the data. When clinical referral data were obtained in PDF format, optical character recognition tools were employed to extract text (21). Referral outcome label preprocessing included manual correction and cleaning, with one study reporting label modifications in up to 10% of cases (22). De-identification of patient information was common (16, 19). Likewise, basic text cleaning steps, such as removing some combination of punctuation, special characters, numbers, stop words, and extra white spaces, were frequently applied (16, 18, 19, 21, 23–25). Stemming or lemmatization to standardize word forms was relatively less frequent (21, 23, 25), and a variety of NLP toolkits were employed for tokenization. Some studies incorporated more advanced preprocessing techniques, including acronym expansion (19), negation detection (25), spelling correction, and synonym replacement for rare clinical terms (24).

Rather than using raw or fully preprocessed free-text, some researchers opted to extract structured entities relevant to the task, such as symptoms or diagnoses (19–21). Input length was also managed either by enforcing maximum word counts (20) or by padding shorter sequences (25). To numerically represent text, a wide range of techniques were employed, including count vectors (e.g., bag-of-words, term frequency–inverse document frequency (TF-IDF) (16, 21, 23–25), word embeddings (e.g., Word2Vec) (24), and subword models like FastText (22). Dimensionality reduction methods, such as Principal Component Analysis (PCA), were infrequently used to optimize these representations (22, 23). While embeddings were often input directly into models, some studies averaged word embeddings to produce a single vector representing the entire referral text (24). Handling of missing data also varied, with some studies excluding incomplete instances (18, 25) or retaining missing fields as-is (20). To enhance dataset size, data augmentation techniques were introduced, including word-swapping models, SMOTE, and other sampling strategies (16, 18, 20).

### NLP triage models

#### Prioritization based on urgency

Three studies prioritized referrals by predicting levels of urgency (21, 23, 25). One of these studies performed multi-class predictions (21), one study performed binary predictions (23) and one study (25) performed both types. Abdel-Hafez et al. (21) grouped otorhinolaryngology referrals into three categories based on urgency. They developed a model using text similarity, specifically Levenshtein distance, between referral texts and clinical prioritization criteria. Other techniques of text similarity tested were compared, specifically Jaccard and cosine. Their best performing model achieved a 0.624 recall and 0.492 precision for highest-urgency referrals, and an overall ‘level of agreement’ of 0.538, with historical clinician-assigned categories.

Tan et al. 2021 (25) developed a convolutional neural network (CNN) to categorize ophthalmology referrals, first into three urgency classes, and then ultimately to binary classes (urgent vs. non-urgent). Additional models tested included artificial neural network (ANN), random forest, and decision tree. When compared to labels determined by senior nurse practitioners, the CNN achieved an overall accuracy of 0.65 on multiclass prediction, and overall AUC and accuracy of 0.83 and 0.81, respectively, for binary predictions. Guzman et al. trained an AdaBoost classifier model to label referrals to spinal surgery as urgent or non-urgent (23). This model outperformed models utilizing *k*-nearest neighbors (k-NN), Naive Bayes, logistic regression, support vector machine (SVM), decision tree, random forest, and XGBoost classifiers. When tested against clinician-generated labels, this model achieved an F1 of 0.531 and an AUC of 0.618.

#### Classification into condition, specialty, or intervention

Six studies developed NLP models to support classification of referrals into condition (18, 21, 24) [although Abdel-Hafez et al. (21) did not report the performance for condition prediction] or specialty/intervention (19, 20, 22). Two of these studies (18, 24) were binary classification tasks, while the remaining studies dealt with multiple outcome classes. Wang et al. (18) developed an ensemble model to classify patients as having inflammatory arthritis vs. non-inflammatory conditions. The ensemble combined a multi-layer BERT model for referral letter text with a light gradient boosting machine model to process blood test data, using a weighted AUC fusion approach to integrate these predictors. Comparator models to process referral letter text utilized different BERT architectures. When evaluated against clinician-confirmed diagnoses following specialist assessment, the ensemble model achieved an AUC of 0.90 and F1 of 0.83. Villena et al. (26) developed a model to classify referrals as conditions that were either covered vs. not covered based on the Chile public health system. The best performing model contained TF-IDF–weighted Word2Vec embeddings and a random forest classifier. This was compared to logistic regression (LR), SVM, and multi-layer perceptron (MLP) classifier models, and achieved a weighted F1 score of 0.85 and AUC of 0.94 when evaluated on a set labelled by three clinical experts.

Aakre et al. (22) utilized a CNN with 300-dimensional FastText embeddings to predict allocation to 28 specialties, using text from self-referral forms. This model outperformed 38 others, including recurrent neural networks and transformer-based models. When evaluated against clinician-assigned labels that were updated post-consultation, this model achieved an AUC of 0.949 and an F1 score of 0.775. Spasic and Button (19) utilized topic distributions from Latent Dirichlet Allocation (LDA) to predict treatment outcomes for adults with hip or knee pain, with 9 outcomes possible such as “Nutritionist”, “Physiotherapy”, and “Surgery”. The best performing model used a k-NN binary classifier trained on a dataset with referral text and Unified Medical Language System mapping, which outperformed topic models trained on referral text alone and a stratified random classifier. Compared to actual treatment outcomes, this model achieved predictive accuracy ranging from 0.5657 (“Review”) to 0.974 (“Nutritionist”). The model by Fudickar et al. (20) classified patients with lower back pain into four treatment categories (rehabilitation, anesthesiology, neurosurgery, or minimal intervention) using an SVM trained on data from self-report questionnaires combined referral texts, which outperformed k-NN and MLP alternatives, achieving an F1 of 0.535.

#### Categorization based on justification

Two studies (16, 24) developed models to assess the level of justification for imaging referrals, classifying into either justified or unjustified. Potocnik et al. (24) classified brain computerized tomography scan referrals using a SVM model with bag-of-words (BoW) representation, custom stop words, and spell correction (BoW + CSW + SC + SVM). This combination outperformed models with other classifiers (LR, random forest), and text representations (TF-IDF). When compared to labels assigned by two experts, this model achieved an AUC of 0.948, weighted accuracy of 0.922, and recall of 0.911. Alanazi et al. (16) developed a BiLSTM model trained on augmented data to classify lumbar spine magnetic resonance imaging referrals. This model was compared to other high-performing deep learning (BiLSTM, CNN), traditional machine learning (random forest, SVM) and traditional statistical (LR) models with varying feature extraction techniques, but the BiLSTM was ultimately selected due to its F1 score of 0.91 and Kappa agreement of 0.56 when compared to two radiologists.

### Feasibility and clinical applicability

Three studies (18, 19, 26) evaluated models on clinical feasibility. Both Wang et al. (18) and Villena et al. (26) included prospective testing of their best performing models as decision-support tools, and Spasic and Button (19) assessed clinician interpretability of their model's outputs. Wang et al. (18) conducted a prospective pilot study that compared performances of their ensemble model (trained on 1,264 historic referral letters) vs. clinicians in labelling 88 patient referrals as inflammatory arthritis vs. not, against the ground truth outcome of clinic diagnosis, and also collected feedback on a decision-support tool. This model outperformed clinicians across all metrics, with a F1 score (mean ± 95% CI) of 0.81 ± 0.08 vs. 0.79 ± 0.09. Additionally, this model demonstrated a potential reduction in time spent for referral assessment by approximately 8 h per week. The developed tool also received positive feedback, due to factors such as increased performance compared to humans and its explainable decision support.

Villena et al. fully implemented their model into clinician workflow in a tertiary hospital, to flag misclassified referrals. This tool identified 87 out of 4,472 referrals that were mislabelled in 7 months. In a qualitative assessment by Spasic and Button (19), two physicians interpreted the topics generated by their model and then rated their similarity in interpretation. This assessment resulted in an inter-annotator agreement of 0.73 (Cohen's kappa) and confirmed high clinical interpretability of model output.

## Discussion

Across multiple outpatient pathways, NLP models demonstrated the potential to significantly enhance the efficiency and effectiveness of triage processes for both binary and multi-class prediction. The included studies described NLP applications that could automate referral prioritization and/or classification, as well as assess justification.

Data preprocessing steps such as cleaning, tokenization, and dimensionality reduction were often tailored to the specific source and structure of referral data and therefore varied considerably across studies. Data augmentation was additionally explored in a minority of papers to improve the quantity of labelled data. The diverse preprocessing and augmentation strategies observed across studies reflects the wide variety of data used to inform referrals. While the lack of standardization limits direct cross-study comparison, the high variability of data captured likely necessitates this variability and demonstrates the applicability of NLP techniques across heterogeneous datasets. The most successful models were able to demonstrate robust performance metrics when benchmarked against clinician and/or expert manually labelled data, including reported AUC values ranging from 0.83 to 0.95, and F1 scores up to 0.91 on their respective tasks. These results suggest that NLP-based triage tools can offer a level of accuracy comparable to manual triage. Reliable support within a time-sensitive process such as triage would greatly improve access to timely care. Although only a minority of studies included prospective deployment of their models, positive outcomes such as improved processing times and increased accuracy were demonstrated in those that did. These pilot studies offer valuable insights into how NLP-based models can realistically be integrated into the triage decision-making process.

LLMs have dominated most recent studies on the application of AI in health, although most studies have been focused on enhancing medical knowledge, making diagnoses and education patients (27). Recent LLMs show dramatic improvements in medical knowledge assessment. Med-PaLM 2 achieved 86.5% accuracy on MedQA (US Medical Licensing Exam-style questions), with physicians preferring its answers to those from other physicians on 8 of 9 clinical axes (28). GPT-4 demonstrated near-similar or better performance compared to disease-specific machine learning models (F1-score ≥85%) for identifying patients with chronic conditions in electronic health records (29). However, for diagnostic tasks, LLMs show promise but generally remain inferior to clinicians (30, 31). Aside from the most common performance metrics (accuracy, comprehensiveness), there remains significant underevaluation of fairness and bias (27), with evidence showing biases related to race and gender, along with privacy risks (32, 33). Recent methodological developments to assess reporting (34–38) and risk of bias (35, 39, 40) of AI in health studies are an important step towards more comprehensive evaluation of this new technology in the medical setting.

This review employed rigorous and transparent methodology to select and evaluate relevant studies, with the aim of synthesizing a comprehensive overview of recent literature. By restricting inclusion to studies published within 5 years of the search, the more current capabilities of triaging applications were prioritized. In addition, the multidisciplinary study team, which included expertise in clinical triage, machine learning, natural language processing, and systematic literature reviews ensured all clinical, technical and methodological aspects were considered. The design of this review was crucial to offer a complete interpretation of model performance, feasibility, and relevance to real-world clinical problems.

We acknowledge that this study has limitations. The literature search included studies published in English only, which may have excluded relevant work conducted in other languages. Additionally, as the pace of research in the application of NLP and AI in healthcare is rapid, the most recent studies that are constantly emerging are not included here. The size and capabilities of transformer-based models have expanded significantly, and we anticipate that the performance of medical triage systems will be enhanced by utilizing secure, locally deployed, open-source transformer models. This study represents the first systematic literature review of studies evaluating current NLP techniques for triage of outpatient referrals, which will need to be regularly updated to ensure continued evaluation of progress in this field. Data synthesis was limited by a high level of inconsistency in reporting of evaluation metrics, dataset allocation, and benchmark comparisons. As a result, meta-analysis was not feasible, and only a narrative synthesis was performed.

Future research would benefit from greater standardization and transparency in reporting. Although variability in data processing and model development is necessary to a certain extent, as mentioned above, standardized evaluation processes are likely feasible. Few studies reported detailed descriptions of inclusion/exclusion criteria, training-validation-test splits, and clinician labeling processes. These details would be crucial to reproduce high-performing models and reliably predict their application to other tasks and/or specialties. Importantly, these studies did not examine potential bias or discrepancies in performance in certain subgroups of patients, which are crucial to ensuring equity in the triaging of limited medical resources, particularly given evidence of inherent bias in medical chart data that may be used for training and evaluating model performance (33). Lastly, as most studies relied solely on retrospective data, further studies focused on prospective effectiveness and clinical impact should be prioritized. As this field of research advances, an increased commitment to methodological rigor, transparent and comprehensive reporting and bias evaluation are critical to ensure trust among stakeholders.

## Conclusion

This review examined 10 studies which developed and evaluated NLP models to support triaging to outpatient specialty care. These models were able to perform tasks such as urgency prioritization, condition classification, and assessment of content. Collectively, the findings from these studies show potential of NLP to improve processing times, clinician workload, and improve accuracy, leading to an improvement in efficiency and quality of care. However, before this promising technology can be safely incorporated into clinical practice, ethical considerations, such as data privacy, model explainability and assessment of bias, must be addressed.
