# Supplementary material for: Systematic literature review and narrative synthesis of the use of natural language processing to triage outpatient referrals
Source: Front Health Serv. 2026 Apr 15;6:1797583. doi: 10.3389/frhs.2026.1797583 (PMC13124730; doi:10.3389/frhs.2026.1797583)
Supplement: Supplementary file 1 [file Datasheet1.pdf]

Supplementary table 1. Search strategies

Date Searched: February 16, 2024		
Database(s): Ovid MEDLINE(R) ALL 1946 to February 16, 2024		
#	Searches	Results
1	artificial intelligence/ or computer heuristics/ or expert systems/ or fuzzy logic/ or exp knowledge bases/ or exp machine learning/ or natural language processing/ or exp neural networks, computer/ or sentiment analysis/	163257
2	(artificial intelligence or AI or GenAI).mp.	105493
3	((large or natural or generative) adj3 (language or text)).mp.	15903
4	(learning adj2 (machine or deep or algorithm*)).mp.	167021
5	((language or transformer or generative) adj2 model*).mp.	8695
6	((sentiment or syntax or semantic) adj analysis).mp.	2677
7	(computational linguistic* or named entry recognition or part of speech tagging or machine translation or speech recognition or text mining or TensorFlow or PyTorch or Word2Vec or GloVe	137286

	or BERT or neural network* or intelligent system* or computer heuristic*).mp.	
8	Generative pre trained transformer*.mp.	287
9	1 or 2 or 3 or 4 or 5 or 6 or 7 or 8	365087
10	Triage/	15299
11	"Referral and Consultation"/	77113
12	(triag* or referral*).mp.	230069
13	10 or 11 or 12	230069
14	9 and 13	2843
15	limit 14 to yr="2019 -Current"	2213
16	limit 15 to english language	2190
17	(Animals/ or Models, Animal/ or Disease Models, Animal/) not Humans/	5164406
18	((animal or animals or canine* or dog or dogs or feline or hamster* or lamb or lambs or mice or monkey or monkeys or mouse or murine or pig or pigs or piglet* or porcine or primate* or rabbit* or rats or rat or rodent* or sheep* or veterinar*) not (human* or patient*)).ti,kf,jw.	2588910
19	17 or 18	5658680
20	16 not 19	2176

21	limit 20 to (comment or editorial or guideline or letter or meta analysis or practice guideline or "review" or "systematic review")	280
22	20 not 21	1896
Date Searched: February 17, 2024		
Database(s): Embase 1974 to 2024 February 16		
#	Searches	Results
1	exp *artificial intelligence/	46090
2	(artificial intelligence or AI or GenAI).mp.	130769
3	((large or natural or generative) adj3 (language or text)).mp.	18320
4	(learning adj2 (machine or deep or algorithm*)).mp.	217686
5	((language or transformer or generative) adj2 model*).mp.	9192
6	((sentiment or syntax or semantic) adj analysis).mp.	2630
7	(computational linguistic* or named entry recognition or part of speech tagging or machine translation or speech recognition or text mining or TensorFlow or PyTorch or Word2Vec or GloVe or BERT or neural network* or intelligent system* or computer	181551

	heuristic*).mp.	
8	Generative pre trained transformer*.mp.	256
9	1 or 2 or 3 or 4 or 5 or 6 or 7 or 8	443165
10	exp *patient triage/	1506
11	*patient referral/	23047
12	(triag* or referral*).mp.	363758
13	10 or 11 or 12	363758
14	9 and 13	4389
15	limit 14 to yr="2019 -Current"	3500
16	limit 15 to english language	3473
17	(animal or animals or canine* or dog or dogs or feline or hamster* or lamb or lambs or mice or monkey or monkeys or mouse or murine or pig or pigs or piglet* or porcine or primate* or rabbit* or rats or rat or rodent* or sheep* or veterinar*).ti,kw,dq,jx. not (human* or patient*).mp.	2222133
18	(exp animal/ or exp juvenile animal/ or adult animal/ or animal cell/ or animal tissue/ or nonhuman/ or animal experiment/ or animal model/) not human/	7513310
19	17 or 18	7589937
20	16 not 19	3453
21	limit 20 to (editorial or letter or note or	431

	"review")	
22	20 not 21	3022
23	limit 22 to (meta analysis or "systematic review")	55
24	22 not 23	2967
25	limit 24 to (conference abstract or conference paper or "conference review")	1011
26	24 not 25	1956
Date Searched: Febrary 17, 2024		
Database: EBSCO CINAHL Plus with Full Text		
**#**	**Searches**	**Results**
S20	S18 NOT S19 Limiters - English Language; Publication Date: 20190101-20241231	773
S19	S16 NOT S17 Limiters - English Language; Publication Date: 20190101-20241231; Publication Type: Anecdote, Commentary, Editorial, Letter, Meta Analysis, Meta Synthesis, Practice Guidelines, Review, Systematic Review	81
S18	S16 NOT S17 Limiters - English Language; Publication Date: 20190101-20241231	854

S17	TI ((animal or animals or canine* or dog or dogs or feline or hamster* or lamb or lambs or mice or monkey or monkeys or mouse or murine or pig or pigs or piglet* or porcine or primate* or rabbit* or rats or rat or rodent* or sheep*) NOT (human* or patient*)) Limiters - English Language; Publication Date: 20190101-20241231	31,080
S16	S9 AND S13 Limiters - English Language; Publication Date: 20190101-20241231	854
S15	S9 AND S13 Limiters - Publication Date: 20190101-20241231	877
S14	S9 AND S13	1,265
S13	S10 OR S11 OR S12	119,879
S12	(triag* or referral*)	105,462
S11	(MH "Referral and Consultation+")	59,051
S10	(MH "Triage")	11,307
S9	S1 OR S2 OR S3 OR S4 OR S5 OR S6 OR S7 OR S8	79,589
S8	Generative pre trained transformer*	25

S7	((computational N1 linguistic*) or "named entry recognition" or "part of speech tagging" or "machine translation" or "speech recognition" or "text mining" or TensorFlow or PyTorch or Word2Vec or GloVe or BERT or (neural N1 network*) or (intelligent N1 system*) or (computer N1 heuristic*))	20,789
S6	((sentiment or syntax or semantic) N1 analysis)	1,267
S5	((language or transformer or generative) N2 model*)	1,373
S4	(learning N2 (machine or deep or algorithm*))	22,032
S3	((large or natural or generative) N3 (language or text))	4,924
S2	(artificial intelligence or AI or GenAI)	29,887
S1	(MH "Artificial Intelligence+")	33,297
Date Searched: Feb. 17, 2024		
Database: Web of Science Core Collection Including: Science Citation Index Expanded (SCI-EXPANDED)--1900-present/Social Sciences Citation Index (SSCI)--1900-present/ Social Sciences Citation Index (SSCI)--1900-present/Conference Proceedings Citation Index – Science (CPCI-S)--1990-present/Conference Proceedings Citation Index – Social Science & Humanities		

(CPCI-SSH)--1990-present		
#	Search Query	Results
1	TS=((artificial intelligence or AI or GenAI)) Editions: WOS.SCI,WOS.SSCI, WOS.ISTP,WOS.ISSHP	209268
2	TS=(((large or natural or generative) NEAR/3 (language or text))) Editions: WOS.SCI,WOS.SSCI, WOS.ISTP,WOS.ISSHP	68063
3	TS=((learning NEAR/2 (machine or deep or algorithm*))) Editions: WOS.SCI,WOS.SSCI, WOS.ISTP,WOS.ISSHP	599984
4	TS=(((language or transformer or generative) NEAR/2 model*)) Editions: WOS.SCI,WOS.SSCI, WOS.ISTP,WOS.ISSHP	63338
5	TS=(((sentiment or syntax or semantic) NEAR/1 analysis)) Editions: WOS.SCI,WOS.SSCI, WOS.ISTP,WOS.ISSHP	25723

6	TS=(((computational NEAR/1 linguistic*) or "named entry recognition" or "part of speech tagging" or "machine translation" or "speech recognition" or "text mining" or TensorFlow or PyTorch or Word2Vec or GloVe or BERT or (neural NEAR/1 network*) or (intelligent NEAR/1 system*) or (computer NEAR/1 heuristic*))) Editions: WOS.SCI,WOS.SSCI, WOS.ISTP,WOS.ISSHP	762756
7	ALL=(Generative pre trained transformer*) Editions: WOS.SCI,WOS.SSCI, WOS.ISTP,WOS.ISSHP	387
8	#7 OR #6 OR #5 OR #4 OR #3 OR #2 OR #1 Editions: WOS.SCI,WOS.SSCI, WOS.ISTP,WOS.ISSHP	1372850
9	TS=((triag* or referral*)) Editions:	160657

	WOS.SCI,WOS.SSCI,<br>WOS.ISTP,WOS.ISSHP	
10	#9 AND #8<br>Editions:<br>WOS.SCI,WOS.SSCI,<br>WOS.ISTP,WOS.ISSHP	2875
11	#9 AND #8 and 2024 or 2023 or 2022 or 2021 or 2020 or 2019 (Publication Years) Editions:<br>WOS.SCI,WOS.SSCI,<br>WOS.ISTP,WOS.ISSHP	2212
12	#9 AND #8 and 2024 or 2023 or 2022 or 2021 or 2020 or 2019 (Publication Years) and English (Languages)<br>Editions:<br>WOS.SCI,WOS.SSCI,<br>WOS.ISTP,WOS.ISSHP	2203
13	#9 AND #8 and 2024 or 2023 or 2022 or 2021 or 2020 or 2019 (Publication Years) and English (Languages) and Review Article or Editorial Material or Letter (Exclude – Document Types)<br>Editions:<br>WOS.SCI,WOS.SSCI,	1992

	WOS.ISTP,WOS.ISSHP	
